# CPL-01, an investigational long-acting ropivacaine, demonstrates safety and efficacy in open inguinal hernia repair

**DOI:** 10.1007/s10029-024-03047-3

**Published:** 2024-05-07

**Authors:** H. T. Xu, J. Zimmerman, T. Bertoch, L. Chen, P. J. Chen, E. Onel

**Affiliations:** 1 Cali (SZ) Biosciences Co., Ltd. Shanghai Branch, Building 7, 690 Bibo Road, Shanghai, R715S China; 2 Cali Biosciences US, LLC, 9675 Businesspark Avenue, San Diego, CA 92131 USA; 3 Trovare Clinical Research, 3838 San Dimas St Ste A280, Bakersfield, CA 93301 USA; 4 CeneExel JBR, 650 East 4500 South, Suite 100, Salt Lake City, UT 84107 USA

**Keywords:** Herniorraphy, long-acting local anesthetic, non-opioid analgesia

## Abstract

**Background:**

There is an unmet medical need for effective nonopioid analgesics that can decrease pain while reducing systemic opioid use. CPL-01, an extended-release injectable formulation of ropivacaine, is designed to safely provide analgesia and reduce or eliminate opioid use in the postoperative period.

**Methods:**

Subjects undergoing open inguinal hernia with mesh were prospectively randomized to 1 of 3 doses of CPL-01 (10, 20, or 30 ml of 2% CPL-01, n = 14, 12, and 14, respectively), Naropin (150 mg, n = 40), or saline placebo (n = 13) infiltrated into the surgical site prior to closure. Pain and rescue medication usage was assessed, and Numeric Rating Scale (NRS) pain scores were adjusted for opioid usage using windowed worst observation carried forward (wWOCF) imputation. The primary efficacy endpoint was the mean area under the curve (AUC) of the NRS pain intensity scores with activity.

**Results:**

Ninety-three subjects were treated, and 91 subjects completed 72 h of post-operative monitoring. Subjects who received the highest dose of CPL-01 in Cohort 3 showed a clinically meaningful reduction in postoperative pain intensity scores, which was the lowest value for any treatment in all cohorts, showing a trend towards statistical significance as compared to the pooled placebo group (p = 0.08), and numerically better than the 40 subjects who received Naropin. Opioid use through 72 h in subjects who received CPL-01 in Cohort 3 was approximately half of that shown in the placebo and Naropin groups; approximately 2/3 of the CPL-01 subjects (9/14) required no opioids at all through the first 72 h after the operation. More CPL-01 subjects avoided severe pain and were ready for discharge earlier than other groups. CPL-01 was safe and well-tolerated, with no clinically meaningful safety signals, and showed predictable and consistent extended-release pharmacokinetics.

**Conclusion:**

Results suggest that CPL-01 may be the first long-acting ropivacaine to address postoperative pain while reducing the need for opioids.

**Supplementary Information:**

The online version contains supplementary material available at 10.1007/s10029-024-03047-3.

## Background

Effective postoperative analgesia is a critical element in patient recovery [1]. Enhanced Recovery After Surgery (ERAS) protocols currently emphasize opioid-sparing or minimizing techniques [2, 3]. Effective multimodal anesthesia, including long-acting local anesthetics, is a key part of the strategy to reduce or eliminate opioid exposure [4, 5]. There is a serious and ongoing unmet medical need for effective nonopioid analgesics that can reduce use of systemic opioids. The use of local anesthetics has been shown to facilitate quicker discharge from post-anesthesia care units (PACUs), due to reductions in opioid-related adverse events, especially postoperative nausea and vomiting [6–8].

While multiple long-acting local anesthetics have been approved, each with different delivery systems (liposomal, collagen scaffolding, sucrose) or combination products (with meloxicam), all of them are bupivacaine based [9]. Ropivacaine differs from bupivacaine in that it is a pure S(-)-enantiomer and not as a racemate, and secondly, ropivacaine is less lipophilic than bupivacaine and is less likely to penetrate large, myelinated motor fibers [10]. Ropivacaine has less cardiovascular and CNS toxicity than racemic bupivacaine in healthy volunteers [10]. Multiple studies have demonstrated that ropivacaine acts more quickly and consistently than bupivacaine, allowing for more optimal dosing before adverse events arise [11–14]. Bupivacaine is more likely to produce symptoms of local anesthetic systemic toxicity (LAST) [15–18].

However, there is no currently available FDA-approved form of long-acting ropivacaine. CPL-01 is an investigational extended release ropivacaine formulation. The ideal pharmacokinetics (PK) for CPL-01 or any long-acting local anesthetic is to achieve steady delivery of the local analgesic over 72 h, allowing consistent pain control no matter what the surgical model, while remaining below the concentrations associated with LAST (2200 ng/mL for ropivacaine, 2000 ng/mL for bupivacaine). The release characteristics of CPL-01 meet these parameters, so consequently CPL-01 could fulfill this significant medical need by safely and effectively managing postoperative pain while decreasing or avoiding opioid exposure, thus reducing the risk of long-term opioid dependence.

This Phase 2b, randomized, double-blind, placebo- and active-controlled, dose escalation study evaluated the safety, efficacy, and pharmacokinetics of a range of doses of CPL-01 in the management of postoperative pain after open inguinal herniorrhaphy compared to placebo and the active control ropivacaine HCl (NAROPIN). Given how commonly it is performed, herniorrhaphy is one of the surgical models endorsed by the United States (US) Food and Drug Administration (FDA) for the testing of novel analgesics [19, 20]. This model is often chosen in drug development to be a surrogate for other soft tissue surgeries [21].

## Methods

This was a phase 2b, randomized, double-blind, placebo- and active-controlled study in subjects between 18–75 years of age undergoing open inguinal herniorrhaphy with mesh under general anesthesia, conducted at 5 study sites in the US.

This study evaluated 3 ascending dose levels of CPL-01, in comparison with Naropin (ropivacaine hydrochloride), or placebo. In each cohort, subjects were randomly assigned in a 3:3:1 ratio to receive either CPL-01, Naropin, or placebo. The use of Naropin as an active control permits a direct comparison and evaluation of the potential benefits provided by the long-acting formulation in CPL-01 to that of conventional, commercially available ropivacaine HCl.

Subjects in Cohort 1 received 10 mL of 2% CPL-01 (~ 200 mg ropivacaine) or 30 mL of 0.5% Naropin (150 mg ropivacaine HCl) or placebo (30 mL 0.9% normal saline). Subjects in Cohort 2 received up to 20 mL of 2% CPL-01 (up to 400 mg ropivacaine) or 30 mL of 0.5% Naropin (150 mg ropivacaine HCl) or placebo (30 mL 0.9% normal saline). Subjects in Cohort 3 received up to 30 mL of 2% CPL-01 (up to 600 mg ropivacaine) or 30 mL of 0.5% Naropin (150 mg ropivacaine HCl) or placebo (30 mL 0.9% normal saline).

Subjects underwent open inguinal herniorrhaphy under general anesthesia and intraoperative analgesia. Hernias were required to be unilateral; there were no other restrictions on the type of hernia. Full inclusion and exclusion criteria are presented in the Supplementary Materials.

Spinal, epidural, or regional anesthesia were not permitted. While many current herniorrhaphy procedures use local anesthesia alone, general anesthesia was used to ensure all subjects began the postoperative period with a similar level of background analgesia, facilitating a more consistent test of the efficacy of the study drug. Near the completion of surgery, a single dose of study drug was administered intraoperatively via local wound infiltration into the surgical site. Approximately one-third to one-half of study drug was placed immediately underneath the aponeurosis of the external oblique, above the inguinal canal (taking care to avoid the nerves) and into the canal itself. Approximately 1/3 of the study drug was placed immediately above the aponeurosis of the external oblique, and the remainder of study drug was placed into the subcutaneous tissue above the level of the fascia.

Intravenous (IV) fentanyl up to 4 μg/kg was permitted for intraoperative pain control. At the start of wound closure each subject received 50 μg IV fentanyl to address the variability in pain perception that can occur as the subject transitions out of general anesthesia. Other intraoperative opioids or any other analgesics (including ketamine), local anesthetics, or anti-inflammatory agents were prohibited, unless needed to treat an adverse event (AE), for pretreatment prior to a needle placement, or to decrease venous irritation.

Following surgery and immediate postoperative recovery, subjects were transferred to the post anesthesia care unit (PACU). Subjects remained in the hospital/research facility for a minimum of 72 h after the start of study drug administration to undergo postoperative assessments. While the postoperative stay is much longer than standard practice, keeping subjects in the hospital allows for more efficient pain score collection with proper use of validated outcome instruments and rescue medication usage accountability, which all contribute to a more robust clinical study. Subjects returned to the study site on Day 7 for follow-up assessments and on Day 28 for the end-of-study (EOS) visit.

Subjects were only able to receive rescue medication upon request for pain control (not prophylaxis), as needed, during the 72-h postoperative period. Postoperative rescue medication consisted of oral (PO) immediate-release oxycodone (no more than 10 mg every 4 h, as needed), IV morphine (no more than 10 mg every 2 h, as needed), and/or PO acetaminophen or PO paracetamol (no more than 1 g [1000 mg] in a 6-h window). The choice of agent was based on the opinion of the blinded assessor. No other analgesic agents, including NSAIDs, were permitted during the 72-h postoperative observation period.

Subjects who were not medically ready for discharge at 72 h were able to receive the same rescue medication as described above to treat postoperative pain until discharge. For subjects who were medically ready for discharge at 72 h, PO acetaminophen/paracetamol (no more than 1000 mg every 6 h, as needed) was recommended for postoperative pain. Subjects were provided with a prescription for oxycodone (up to 10 mg PO every 4 h [q4h] as needed) only if they had received 10 mg or more of oxycodone in the 12 h prior to discharge. Pain medication usage post discharge was recorded in a patient diary.

Postoperative pain was assessed using the validated Numeric Rating Scale (NRS) [5]. The NRS is a validated outcomes instrument, commonly used in studies of new pain medications, in which a blinded assessor asks the patients to rate the intensity of their pain on a scale from 0 (no pain) to 10 (worst pain imaginable). Pain was assessed with activity (NRS-A); activity was defined as sitting up from a supine or recumbent position. The primary efficacy endpoint was the mean area under the curve (AUC) of the NRS-A pain intensity scores from 0–72 h (AUC_0-72_) of CPL-01 compared to pooled placebo. Pain scores were adjusted for the use of opioid rescue medication using windowed worst observation carried forward (wWOCF) imputation. NRS pain intensity scores at rest (NRS-R) were also assessed in the same manner, using wWOCF.

Other secondary endpoints included time to first opioid use, total opioid use in IV morphine equivalent doses (MED), time spent in the PACU, and discharge readiness based on the Modified Postanesthetic Discharge Scoring System (MPADSS).

Safety assessments included adverse events (AEs), potential Local Anesthetic Systemic Toxicity (LAST) symptoms, clinical safety laboratory tests, vital signs, ECGs and wound healing. Blood samples for determination of ropivacaine plasma concentrations were collected prior to study drug administration, throughout the 72-h postoperative period, and on Day 7 during the outpatient visit. Plasma concentrations of ropivacaine were determined by means of a validated liquid chromatography with tandem mass spectrometry (LC–MS/MS) assay.

### Ethical statement

The original protocol and amendment, informed consent form (ICF), and all other written documents provided to subjects were reviewed and approved by the Advarra Institutional Review Board (IRB) prior to initiation of the study. All subjects signed the ICF prior to any study procedures. This trial was designed and conducted in accordance with US and international standards of Good Clinical Practice (GCP) (FDA regulations 21 CFR 312 for IND studies and International Council for Harmonisation of Technical Requirements for Pharmaceuticals for Human Use [ICH] Guideline E6).

### Statistical methods

The primary endpoint was assessed for all subjects who were randomized and treated with study drug as assigned (the full analysis set [FAS]). Safety was assessed in all subjects who were randomized and treated with study drug as received (the safety analysis set [SAS]). Due to the small sample size of the study, normality assumptions were assessed using a Shapiro–Wilk test. Results informed the use of a non-parametric test, Wilcoxon Rank Sum, for the NRS endpoints.

Pharmacokinetic parameters were calculated by noncompartmental methods for each treatment: AUC0-last, AUC0-∞, Cmax, Tmax, λz, and t1/2. Safety results were summarized descriptively by treatment group for the SAS.

### Subject population and disposition

**Fig. 1:**
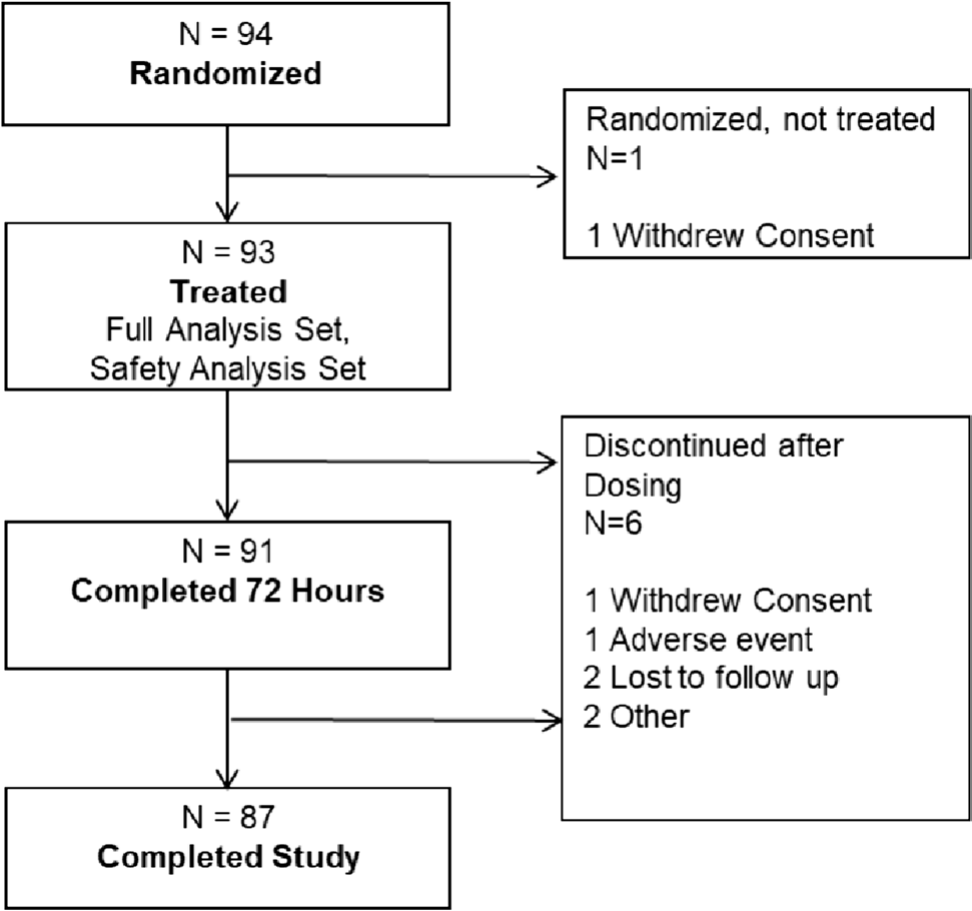
Patient disposition

Disposition and analysis populations are summarized in Fig. 1. Demographics were similar between groups (Supplemental Table 1). Overall, 93.4% of the population were white, 93.4% were male (typical for this procedure), and the mean age was 47 years old.

Surgical parameters were comparable across treatment groups. Procedures were comparably distributed between left (45%) and right (55%) sides. The majority of surgeries used polypropylene mesh (65%) and types of mesh were balanced across groups. Surgeries required an average of 60 min to complete (range 22 to 135 min). All subjects were medically ready for discharge at 72 h.

## Results

Overall, results demonstrated that CPL-01 controlled postoperative pain in a dose-dependent manner, compared to placebo and the active control Naropin, identifying an effective dose of CPL-01 that provided analgesia and reduced opioid usage in a clinically meaningful manner.

### AUC of NRS pain scores

The primary efficacy endpoint was the mean AUC_0-72_ of the NRS-A pain intensity scores using wWOCF of CPL-01 compared to pooled placebo, performed on the FAS. Results for all groups are presented in Table 1.

**Table 1:** Pain intensity during the postoperative period, as measured by the AUC_0-72_ of the NRS-A pain intensity scores using wWOCF(Full analysis Set)

	Pooled Placebo (N = 13)	Pooled NAROPIN (N = 40)	CPL-01		
			Cohort 1 10 mL of 2% CPL-01 (N = 14)	Cohort 2 20 mL of 2% CPL-01 (N = 12)	Cohort 3 30 mL of 2% CPL-01 (N = 14)
Mean (SD) (SE)	369.2 (133.41) (37.00)	322.5 (176.63) (27.93)	319.9 (107.92) [28.84]	306.3 (140.98) [40.70]	286.8 (113.70) (30.39)
p-value^1^ vs. Pooled Placebo			0.2159	0.2210	0.0805

*AUC*_*0-72*_ area under the curve from time 0 to 72 h, *NRS-A* Numeric Rating Scale with activity, *wWOCF* windowed worst observation carried forward, *SE* standard error, *SD* standard deviation

^1^ Statistics reflect results of a Wilcoxon rank-sum test comparing CPL-01 with the control (pooled placebo, cohort-specific NAROPIN, or pooled NAROPIN).

**Fig. 2:**
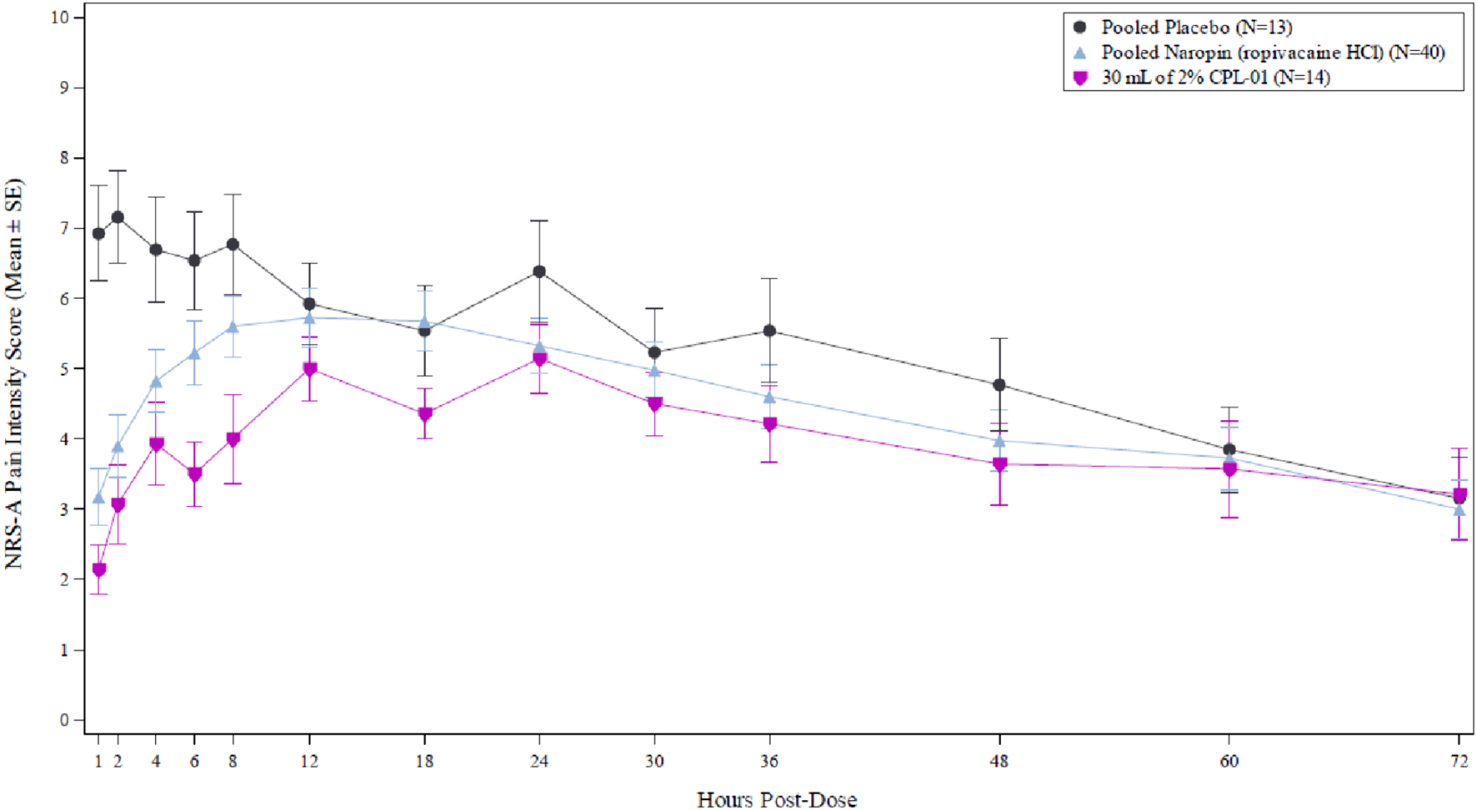
Mean (SE) NRS-A Pain Intensity Scores Through 72 Hours Post-Dose Using wWOCF, Full Analysis Set, Cohort 3

Subjects who received the highest dose of CPL-01 in Cohort 3 had a mean (SD) AUC_0-72_ of 286.8 (113.70), which was the lowest value for any treatment group in all cohorts, showing a trend towards significance as compared to the pooled placebo group (mean AUC_0-72_ of 369.2, p = 0.08) and numerically lower than the mean (SD) AUC_0-72_ of 322.5 (176.63) for the pooled NAROPIN group. The difference was not statistically significant, likely due to the small sample size of this study. The mean NRS-A pain intensity scores using wWOCF over 72 h for Cohort 3 as compared to pooled placebo and pooled Naropin are shown in Fig. 2.

Clinically, a difference in AUC greater than 1 point per hour of the AUC time period between two treatment groups is generally considered to be clinically meaningful [22]. For example, a difference in AUC greater than 72 between two treatment groups for AUC_0-72_ would suggest clinical benefit for the treatment group with the lower AUC compared to the treatment group with the higher AUC. Subjects in Cohort 3 showed a mean 82.4-point difference in the AUC_0-72_ as compared to pooled placebo, demonstrating that the statistical trend was also clinically meaningful.

### Severe pain

**Fig. 3:**
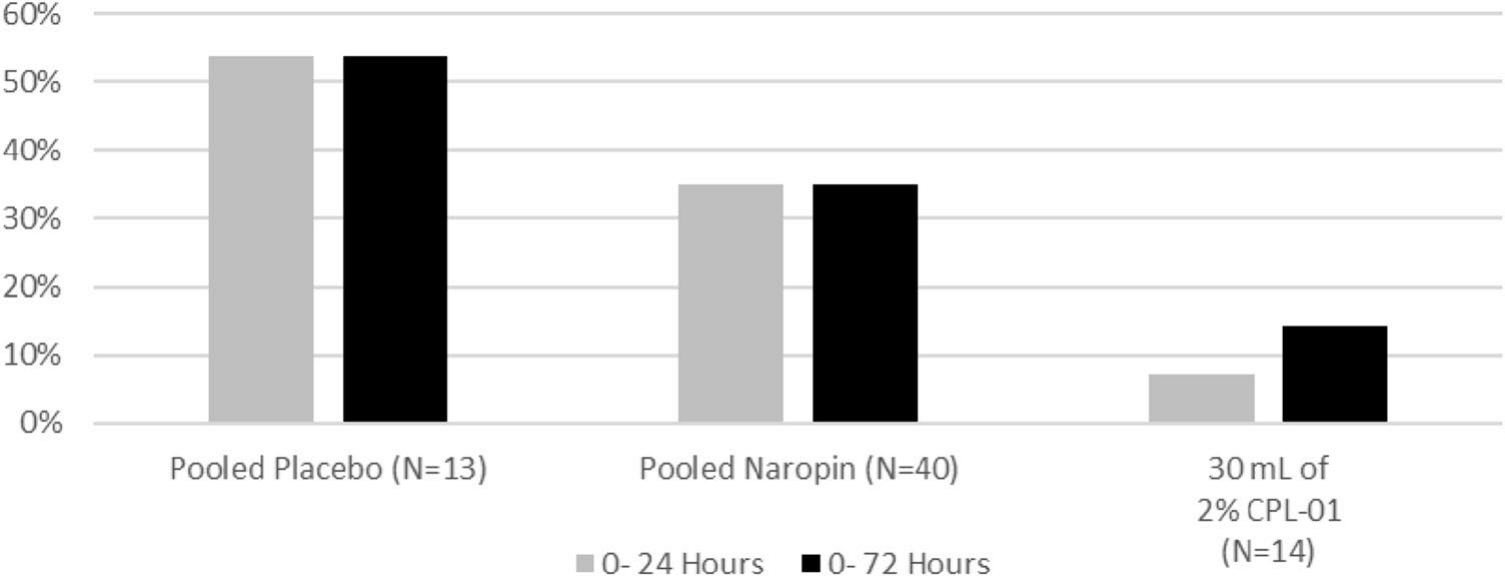
Subjects with severe pain during the postoperative period

An NRS score of greater than or equal to 7 is considered severe pain, and opioids are indicated to treat severe pain. Overall, fewer subjects who received CPL-01 experienced severe pain compared to the pooled placebo group or the pooled NAROPIN group (Fig. 3).

In the first 24 h, 1 subject (7.1%) who received CPL-01 in Cohort 3 experienced severe pain (NRS-R score ≥ 7), as compared to 7 (53.8%) in the pooled placebo group, and 14 (35.0%) in the pooled NAROPIN group. Over the first 72 h, one additional subject (total of 2; 14.3%) who received CPL-01 in Cohort 3 experienced severe pain, as compared to 7 (53.8%) in the pooled placebo group, and 14 (35.0%) in the pooled NAROPIN group.

Over the first 72 h, fewer subjects who received CPL-01 in Cohort 3 experienced severe pain compared to the pooled placebo group or the pooled NAROPIN group.

### Opioid use

Rescue mediation was provided upon request for pain control. Subjects who received CPL-01 in all cohorts had numerically lower mean total opioid rescue mediation use (in morphine equivalent doses [MED]) than the pooled placebo and Naropin groups in the 72-h postoperative period. Total opioid rescue mediation use in all groups is summarized for key time periods in Supplemental Table 2).

**Fig. 4:**
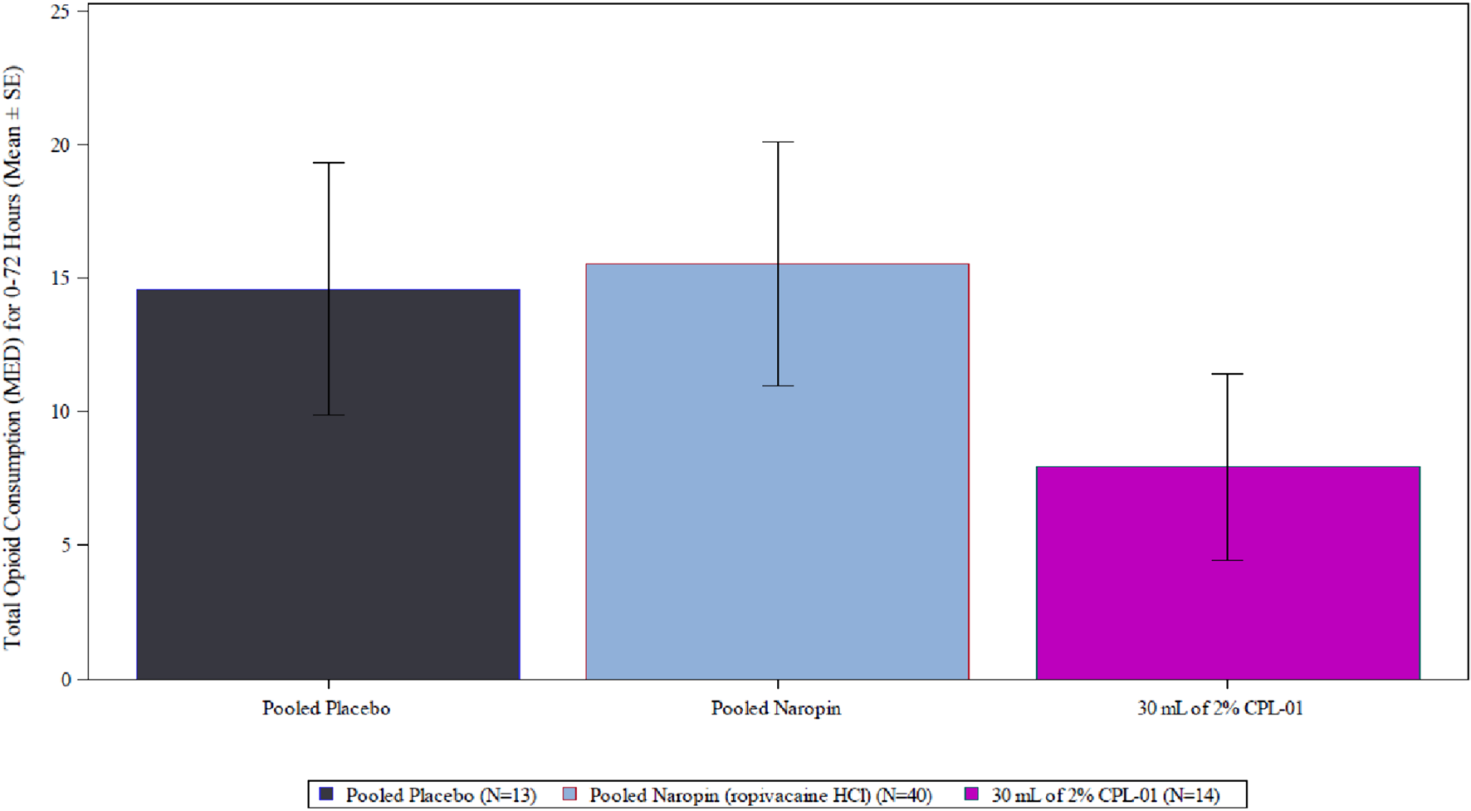
Mean (SE) Total Opioid Consumption (MED) for 0–72 Hours, Full Analysis Set, Cohort 3 CPL-01 with Pooled Placebo and Pooled Naropin

In Cohort 3, the mean (SD) total MED over the first 72 h was 7.93 (13.019), which was approximately half the values observed in the pooled placebo and Naropin groups, of 14.58 (17.037), and 15.53 (28.815), respectively (Fig. 4).

In the first 24 h, subjects who received CPL-01 in all cohorts had numerically lower mean MED than cohort specific placebo and Naropin groups, and pooled placebo and Naropin groups.

A greater proportion of subjects in the CPL-01 treatment groups remained opioid-free during the first 72 h: 70% (28/40) subjects in all CPL-01 treatment groups, as compared to 30.8% of the pooled placebo group and 52.5% of the pooled Naropin group.

In general, CPL-01 treatment delayed the onset of opioid usage, and rates of usage remained lower overall. Through 24 h, the probability of a subject receiving opioid rescue medication was 69.2% in the pooled placebo group, 45.3% in the pooled NAROPIN group, and 35.7% in CPL-01 Cohort 3. Similarly, through 72 h, the probability of a subject receiving opioid rescue medication was 69.2% in the pooled placebo group, 47.9% in the pooled NAROPIN group, and 35.7% in CPL-01 Cohort 3.

Through Day 7, subjects who received CPL-01 in all cohorts received numerically less mean total MED than the pooled placebo and NAROPIN groups. In subjects who received CPL-01 in Cohort 3, the mean (SD) total MED through Day 7 was 12.2 (21.46), compared to values observed in the pooled placebo and NAROPIN groups of 15.8 (20.06) and 21.9 (41.40), respectively. The trends were similar for opioid usage through Day 28.

### Time to discharge

**Fig. 5:**
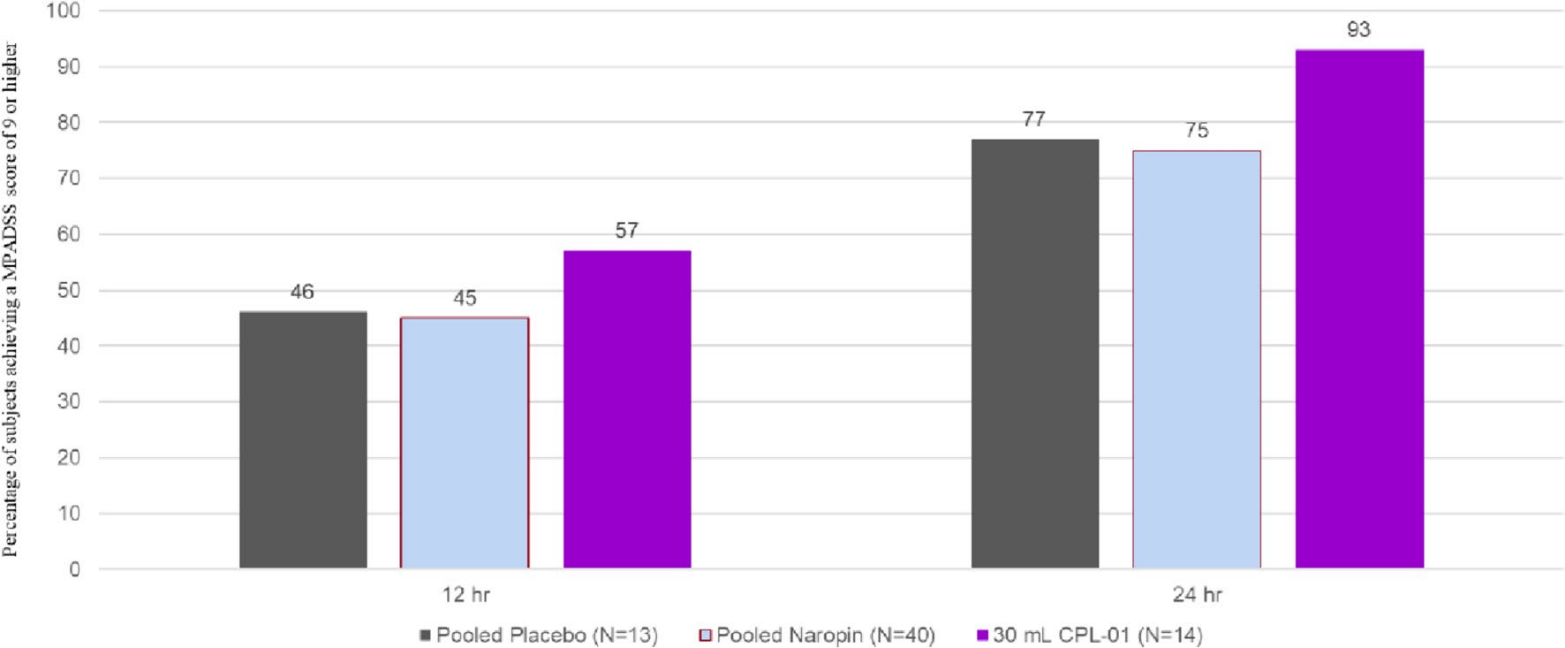
Percentage of subjects ready for discharge at 12 h and 24 h, Full Analysis Set

Discharge readiness was assessed using the MPADSS, which scores five components: vital signs, ambulation, nausea and vomiting, pain, and surgical bleeding. A total MPADSS score (sum of all components) of 9 or higher was considered ready for discharge. The percentage of subjects achieving a MPADSS score of 9 or higher during the postoperative period is presented in Fig. 5 for subjects in Cohort 3, as compared with pooled placebo and pooled Naropin. At 12 h and at 24 h, a notably greater percentage of subjects who received CPL-01 in Cohort 3 were ready for discharge, as compared to the pooled placebo and pooled Naropin groups.

**Fig. 6:**
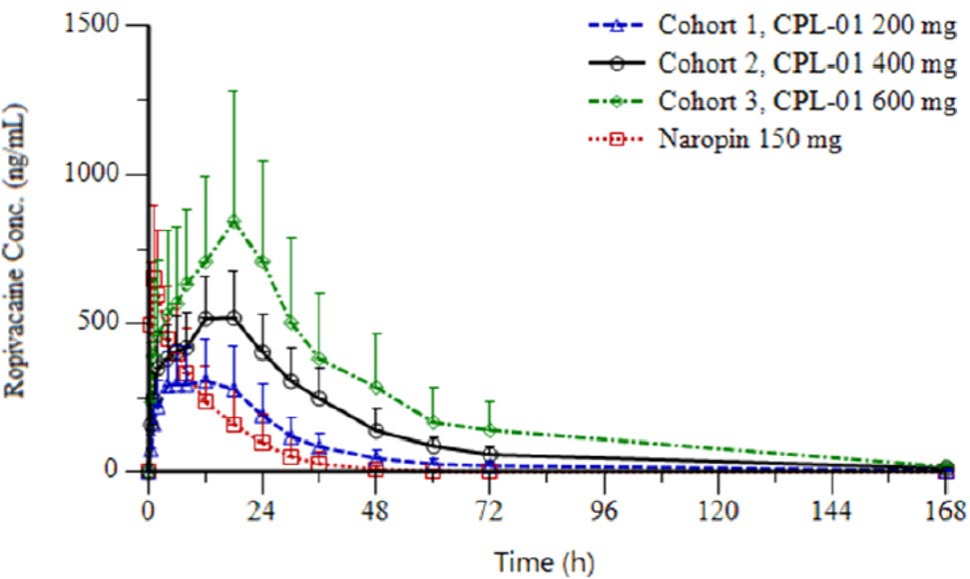
Mean Ropivacaine Plasma Concentrations Over Time

### Pharmacokinetics

The PK of CPL-01 was characterized by consistent and predictable exposure, demonstrating the extended-release formulation. （Fig. 6) Select PK parameters are shown in Table 2.

**Table 2:** Plasma Ropivacaine Pharmacokinetic Parameters

	Cohort 1 10 mL of 2% CPL-01 (N = 14)	Cohort 2 20 mL of 2% CPL-01 (N = 12)	Cohort 3 30 mL of 2% CPL-01 (N = 14)	Pooled NAROPIN 150 mg n = 40
T_max_, (hr) median (range)	7.9 (3.7–18.0)	14.9 (5.6–23.8)	17.8 (1.0–23.8)	0.9 (0.3–17.8)
C_max_ (ng/mL) GeoMean (CV%)	343 (40.4)	556 (25.7)	836 (42.2)	652 (37.6)
Max C_max_ (ng/mL)	697	838	1880	1220
AUC_0-last_ (h*ng/mL) GeoMean (CV%)	9520 (37.5)	20,100 (29.9)	32,500 (47.2)	6750 (46.9)
AUC_0-24_ (h*ng/mL) GeoMean (CV%)	5950 (34.7)	10,300 (22.5)	14,800 (42.7)	6170 (40.3)
AUC_0-∞_ (h*ng/mL) GeoMean (CV%)	9,770 (37.0)	20,700 (29.1)	33,400 (47.1)	6800 (46.4)
Half life (h) GeoMean (CV%)	41.3 (25.3)	33.3 (28.9)	34.2 (19.8)	4.75 (40.1)

*AUC*_*0-∞*_ area under the concentration time curve from time 0 extrapolated to infinity, *AUC*_*0-last*_ area under the concentration time curve from time 0 to last measured timepoint, *AUC*_*0-24*_ area under the concentration versus time curve from time 0 to 24 h, *C*_*max*_ maximum concentration, *CV%*  coefficient of variation, *GeoMean*  geometric mean, *Max*  maximum *T*_*max*_ time to maximum concentration

Ropivacaine plasma concentration in subjects administered NAROPIN reached the median T_max_ at less than 1 h; in subjects administered CPL-01, the highest observed plasma concentration (1880 ng/mL) was observed in Cohort 3 18 h after administration. CPL-01 exhibited a longer t_½_ than NAROPIN/ropivacaine HCl.

CPL-01 demonstrated systemic delivery of ropivacaine that was generally 3 times longer when compared to a given dose of NAROPIN/ropivacaine HCl. Where NAROPIN delivered 90.6% of its ropivacaine dose in the first 24 h, 600 mg of CPL-01 delivered 44.3% of its ropivacaine dose within the first 24 h.

Exposure showed a consistent dose-dependent effect in the CPL-01 dose groups. Overall ropivacaine exposure, as measured by AUC_0-last_, was greater in all CPL-01 dose groups than in the NAROPIN/ropivacaine HCl groups. For CPL-01 to deliver > 90% of the ropivacaine total exposure based on AUC_0-∞_ it generally required 72 h or longer, and showed consistent and predictable delivery over time.

### Safety

There were no clinically meaningful or dose-dependent safety signals. The overall incidence in treatment-emergent adverse events (TEAEs) was similar across treatment groups and typical of common perioperative side effects (Supplemental Table 3). The incidence of TEAEs ranged from 64.3% to 83.3% of subjects who received CPL-01 by cohort, 76.9% of subjects who received placebo, and 72.5% of subjects who received Naropin.

Most TEAEs were mild to moderate in severity. There were only 2 subjects who reported TEAEs that were severe. Six subjects reported TEAEs that were suspected to be related to Naropin. No events considered to be related to CPL-01.

Common TEAEs in CPL-01-treated subjects across all cohorts were constipation, headache, dizziness, nausea, and vomiting. In CPL-01-treated subjects in Cohort 3 TEAEs that occurred most frequently were constipation (43%), headache (21%), and dizziness (21%). Nausea and vomiting occurred in 2 subjects (14.3%) each.

One subject in the Naropin group discontinued the study due to an TEAE, and one subject in the CPL-01 Cohort 2 group experienced 2 SAEs, both unrelated to treatment. There were no deaths.

Wound healing was normal by Southampton Wound Scoring (0 or I) at every timepoint for all but 2 subjects. No subjects had Southampton Wound scores classified as major (Score IV or V).

Potential LAST-related TEAEs occurred in 10/40 (25.0%) of subjects who received NAROPIN, 5/40 (12.5%) of subjects who received CPL-01, and no subjects who received placebo. Potential LAST-related TEAEs in CPL-01-treated subjects included dysgeusia (1 subject), dizziness (3 subjects) and muscle twitching (1 subject). All of these events were mild or moderate in severity, and none of these events was associated in time with the peak plasma ropivacaine concentration, nor did any of these subjects have a peak plasma concentration > 840 ng/mL, making a diagnosis of LAST unlikely.

## Conclusion and discussion

Many patients receive their first exposure to opioids following surgery, and postoperative exposure of as few as 3 days can increase the risk of chronic opioid use [23, 24]. Persistent usage carries the risk of dependence, addiction, and abuse. Current estimates of the economic burden of the opioid crisis, including increased health care costs, productivity loss, and support from services such as law enforcement, exceed $100 billion per year. [25, 26].

Inguinal hernia repair is one of the most common surgeries, with an estimated 800,000 procedures reported each year in the US [27]. Herniorrhaphy reliably produces persistent pain symptoms typically lasting over 72 h from the time of surgery*.* Open inguinal hernia repair is believed to cause greater postoperative pain than minimally invasive techniques [28], and consequently, patients are often prescribed more opioids at discharge [29]. In spite of the generally accepted societal goal of opioid reduction, between 36 and 90% of inguinal herniorrhaphy patients are still prescribed opioids at discharge [29–31]; 1.5% of previously opioid-naïve patients were continuing to refill opioid prescriptions a full 3 months after surgery [32]. Further, more opioids are prescribed than actually used by patients in hernia [28, 30, 31, 33], and this did not differ significantly by surgical approach [29], indicating an opportunity for a reduction in opioid prescribing across the different techniques. Notably, patients who had herniorrhaphy performed using local anesthetic and general anesthesia were significantly more likely to avoid opioids [28].

In this study, CPL-01 was associated with decreased pain and opioid usage, demonstrating a dose-proportional effect on the NRS-A with pain intensity decreasing with increasing CPL-01 dose. In Cohort 3, 14 subjects who received CPL-01 showed a mean AUC through 72 h of wWOCF adjusted NRS-A (the primary endpoint) that showed a trend towards significance as compared to the 13 subjects who received placebo (p = 0.08) and numerically better than the 40 subjects who received Naropin. These pain differences were considered to be clinically meaningful because they exceeded more than one AUC point per hour [22]. Subjects who received CPL-01 were less likely to experience severe pain in the post-operative period, as measured at rest (AUC_0-72_ of NRS-R), the state in which subjects spend the majority of their recovery time.

Subjects who received CPL-01 also showed substantial reduction in the need for opioids. Opioid use through 72 h in subjects who received CPL-01 in Cohort 3 was approximately half of that shown in the placebo and Naropin groups. Approximately 2/3 of the CPL-01 subjects (9/14) required no opioids at all through the first 72 h after the operation, compared to roughly half of the Naropin subjects and 30% of the placebo subjects. These differences are clinically meaningful for subjects in the hospital. By reducing somnolence and other opioid-related effects that may interfere with surgical rehabilitation, patients should be able to recover full function more quickly; by reducing urinary retention and respiratory depression, patients should be able to avoid interventions such as catheterization and intubation. Reducing opioids needed at discharge may also reduce the amount of unused opioids available for diversion and misuse by the general population.

Ropivacaine was specifically developed as a safer local anesthetic alternative to bupivacaine; with a higher dose tolerated and fewer cardiac impacts [21]. The safety profile of CPL-01 observed in this study is consistent with the safety of ropivacaine in general. There were no clinically meaningful or dose-dependent safety signals, or signs of LAST.

Overall, the PK of CPL-01 was consistent with the extended-release formulation. The mean maximum concentrations were in an acceptable range for all 3 CPL-01 dose levels, with no indication of either “dose-dumping” or concentrations approaching the threshold for LAST (2,200 ng/mL for ropivacaine, 2000 ng/mL for bupivacaine). Overall ropivacaine exposure was greater in all CPL-01 dose groups than in the Naropin group. CPL-01’s consistent and predictable delivery over the postoperative period in multiple surgical models may give practitioners greater confidence in the safety and efficacy of CPL-01. If surgeons are confident that their patients’ pain will be well-controlled over time, they will be more likely to reduce opioid prescribing.

Open inguinal hernia was chosen to test CPL-01 because it is an accepted surgical model intended to act as surrogate for other pain states [19, 20], from which the efficacy of the local analgesic can be generalized [25, 26]. In clinical practice, minimally invasive inguinal hernia repair and early postoperative discharge are common practices. However, to adequately test the efficacy of CPL-01, certain aspects of the protocol (general anesthesia, a 72-h postoperative stay, etc.) were mandated prior to review and agreement by the US FDA. Taking these steps maximized the scientific rigor and generalizability of the study results.

In conclusion, while the sample size was small, the safety and efficacy results based on the primary and secondary endpoints indicate that the objectives of the study were met. CPL-01 was shown to numerically, but meaningfully, reduce postoperative pain intensity scores, including severe pain for which opioids would be prescribed, and to reduce or avoid opioid usage during the initial 72-h postoperative period as compared to placebo and NAROPIN®, the currently marketed form of ropivacaine HCl injection. If Phase 3 studies confirm similar safety and efficacy, then CPL-01 may provide a novel extended-release formulation of ropivacaine for surgeons to provide improved analgesia and reduce opioid usage with improved patient safety.

### Supplementary Information

Below is the link to the electronic supplementary material.

Supplementary file1 (DOCX 35 KB)

## Data Availability

The data that support the findings will be available in Clinical Trials at https://clinicaltrials.gov/study/NCT05080959 following an embargo from the date of publication to allow for commercialization of research findings.
