# Supplementary material for: CPL-01, an investigational long-acting ropivacaine, demonstrates safety and efficacy in open inguinal hernia repair
Source: Hernia. 2024 May 7;28(4):1345–54. doi: 10.1007/s10029-024-03047-3 (PMC11297089; doi:10.1007/s10029-024-03047-3)
Supplement: Supplementary file 1 — Supplementary file1 (DOCX 35 KB) [file 10029_2024_3047_MOESM1_ESM.docx]

Supplemental Material

Inclusion and Exclusion Criteria

**Inclusion Criteria**

For inclusion into the trial, each subject was to have met all of the following criteria:

1. Was willing and able to sign the informed consent form (ICF) prior to study participation.
2. In the medical judgment of the Investigator, was a reasonably healthy adult 18 - 75 years of age, inclusive, and American Society of Anesthesiology (ASA) physical Class 1 or 2 at the time of randomization (Clinical Study Protocol Appendix 1 [Appendix 16.1.1]).
3. Planned to undergo an elective open inguinal herniorrhaphy with mesh under general anesthesia without collateral procedures or additional surgeries. Endotracheal intubation was not required.
4. Had a body mass index ≤ 39 kg/m^2^.
5. If female, must have met all of the following:
   1. Females of child-bearing potential must have had a negative serum pregnancy test at Screening and negative urine pregnancy test at check-in before surgery;
   2. No plan to become pregnant or to breast feed during the study;
   3. Was surgically sterile or at least 1 year post-menopausal, had a monogamous partner who is surgically sterile, had a same sex partner, or (one of the following must apply):
   - was practicing double-barrier contraception.
   - was practicing abstinence (must agree to use double-barrier contraception in the event of sexual activity). Site personnel provided instructions on what is an acceptable method.
   - was using an insertable, injectable, transdermal, or combination oral contraceptive approved by the FDA for at least 2 months prior to Screening and committed to the use of an acceptable form of birth control while participating in the study.
6. If male, unless the subject has a same sex partner, was either sterile (surgically or biologically) or committed to an acceptable double barrier method of birth control until at least 30 days after study drug administration. Site personnel provided instructions on what is an acceptable method.
7. In the judgment of the Investigator, was willing and able to complete study procedures and pain scales and to communicate meaningfully with study personnel and return for outpatient follow-up visits as required.

**Exclusion Criteria**

Any of the following was regarded as a criterion for exclusion from the trial:

1. Had previously undergone inguinal herniorrhaphy (on either side) with the exception of a pediatric herniorrhaphy prior to 2 years of age.
2. Had undergone 3 or more surgeries within 12 months prior to signing the ICF, other than for diagnostic procedures (e.g., colonoscopy).
3. In the opinion of the Investigator, subject
   1. Had a concurrent painful condition, including frequent migraines or other headaches, that may have required analgesic treatment during the study period or may have confounded postsurgical pain assessments.
   2. Had active skin disease or other clinically significant abnormality at the anticipated surgical that could have interfered with the planned surgery.
4. Had known hypersensitivity or known allergy, as determined by the Investigator, to ropivacaine, sesame oil, soybeans, fentanyl, oxycodone, morphine, acetaminophen, or the inactive ingredients (i.e., excipients) of the study drug or any peri- or postoperative medications used in this study.
5. As determined by the Investigator (with input from the study’s Medical Monitor if requested by the Investigator), subject had a history or clinical manifestation of significant medical, neuropsychiatric, or other condition including a clinically significant existing arrhythmia, bundle branch block or abnormal ECG, myocardial infarction or coronary arterial bypass graft surgery within the prior 12 months, significant abnormal clinical laboratory test value, or known bleeding abnormality that could have precluded or impaired study participation or interfered with study assessments.
6. Had a history of malignant hyperthermia or glucose-6-phosphate dehydrogenase deficiency.
7. Had history or evidence of impaired liver function (e.g., ALT > 3 × upper limit of normal [ULN] or total bilirubin > 2 × ULN), active hepatic disease, or cirrhosis.
8. Had history or evidence of impaired renal function (e.g., creatinine > 1.5 × ULN).
9. Had a history of malignancy in the past year, with the exception of nonmetastatic basal cell or squamous cell carcinoma of the skin or localized in situ carcinoma of the cervix.
10. Had active COVID-19 infection within 3 months prior to surgery.
11. Had a history of, or positive test results for human immunodeficiency virus, hepatitis B surface antigen, or hepatitis C virus antibody at Screening.
12. The following were considered disallowed:
    1. Concurrent use of potent CY1A2 inhibitors, such as cimetidine, enoxacin, fluvoxamine, ciprofloxacin, gemifloxacin, levofloxacin, moxifloxacin, norfloxacin, or ofloxacin.
    2. Within 24 hours prior to the scheduled surgery, had taken any opioid
    3. Within 3 days prior to the scheduled surgery, had taken a long-acting opioid
    4. Within 5 days prior to the scheduled surgery, had received bupivacaine or ropivacaine
    5. Had known or suspected daily use of opioids for 7 or more consecutive days within the previous 6 months.
    6. Within 7 days prior to the scheduled surgery, had taken any CNS active agent as an analgesic adjunct medication, such as anticonvulsants, gabapentinoids, antidepressants (such as serotonin and norepinephrine reuptake inhibitors [SNRIs], selective serotonin reuptake inhibitors [SSRIs], and tricyclic antidepressants), benzodiazepines, sedative-hypnotics, clonidine and other central alpha-2 agents (e.g., tizanidine), ketamine, or muscle relaxants.
    - These drugs were permitted if prescribed for non-pain indications and the dose has been stable for at least 30 days prior to surgery. The dose must have remained stable throughout the study.
    - Use of benzodiazepines and non-benzodiazepines (eszopiclone, ramelteon, zaleplon and zolpidem) was permitted to treat insomnia during the postoperative period.
    1. Within 7 days prior to the scheduled surgery and throughout the study, had taken antiarrhythmics except beta-blockers, digoxin, warfarin (see exception below), lithium, or aminoglycosides or other antibiotics for an infection (ophthalmic use or for treatment or prophylaxis of postoperative surgical site infections is permitted).
    2. Within 14 days prior to the scheduled surgery and throughout the inpatient period, had taken or using any cannabidiol-containing products, dietary supplements, or overthe-counter (OTC) preparations (e.g., chaparral, comfrey, germander, jin bu huan, kava, pennyroyal, skullcap, St. John’s wort, or valerian).
    3. Within 28 days prior to the scheduled surgery, had received parenteral or oral corticosteroid treatment (steroid inhaler for allergy or asthma treatment, topical steroid for a non-clinically significant skin condition not involving the area of surgery or ophthalmic steroids are permissible).
    4. Was taking an antianginal, antihypertensive agent or diabetic regimen at a dose that has not been stable for at least 30 days or which was not expected to remain stable while participating in the study.
13. In the opinion of the Investigator, within the past year had a history of illicit drug use or prescription medicine or alcohol abuse (regularly drinks > 4 units of alcohol per day, where 1 unit = 8 ounces beer, 3 ounces of wine, or 1 ounce of spirits).
14. Had a positive alcohol breath/saliva test result indicative of alcohol use, or a positive urine drug screen result indicative of illicit drug use (unless results can be explained by a current prescription or acceptable OTC medication at Screening as determined by the Investigator) at Screening and/or prior to surgery.
15. Had previously participated in a clinical study with CPL-01.
16. Had participated in another clinical trial or used an investigational product within 30 days or 5 half-lives, whichever is longer, prior to the planned surgery or was scheduled to receive any other investigational product while participating in the study.

**Supplemental Table 1. Demographic Characteristics, Efficacy Evaluable Set**

|  | | | **Cohort 1** | | | **Cohort 2** | | | **Cohort 3** | | |  |
| --- | --- | --- | --- | --- | --- | --- | --- | --- | --- | --- | --- | --- |
|  | **Pooled Placebo (N=13)** | **Pooled Naropin (N=38)** | **Placebo (N=4)** | **Naropin (ropivacaine HC1) (N=13)** | **10 mL of 2% CPL-01 (N=14)** | **Placebo (N=4)** | **Naropin (ropivacaine HC1) (N=11)** | **20 mL of 2% CPL-01 (N=12)** | **Placebo (N=5)** | **Naropin (ropivacaine HC1) (N=14)** | **30 mL of 2% CPL-01 (N=14)** | **Total**  **(N=91)** |
| Age (years) |  |  |  |  |  |  |  |  |  |  |  |  |
| n | 13 | 38 | 4 | 13 | 14 | 4 | 11 | 12 | 5 | 14 | 14 | 91 |
| Mean (SD) | 43.1 (14.74) | 50.2 (13.17) | 41.3 (11.62) | 50.8 (15.10) | 38.7 (11.80) | 36.3 (14.52) | 43.1 (12.32) | 44.0 (14.93) | 50.0 (16.81) | 55.2 (9.80) | 51.2 (13.76) | 46.8 (14.00) |
| Median (Q1, Q3) | 45.0 (31.0, 56.0) | 53.0 (41.0, 60.0) | 39.0 (32.0, 50.5) | 53.0 (40.0, 62.0) | 38.0 (32.0, 48.0) | 35.5 (24.0, 48.5) | 43.0 (29.0, 55.0) | 45.0 (31.5, 57.5) | 59.0 (36.0, 62.0) | 56.5 (45.0, 62.0) | 53.0 (48.0, 61.0) | 49.0 (36.0, 59.0) |
| Min, Max | 22, 65 | 25, 74 | 31, 56 | 25, 74 | 22, 62 | 22, 52 | 25, 59 | 21, 63 | 28, 65 | 41, 73 | 20, 65 | 20, 74 |
| Sex |  |  |  |  |  |  |  |  |  |  |  |  |
| Male | 13 (100%) | 36 (94.7%) | 4 (100%) | 11 (84.6%) | 11 (78.6%) | 4 (100%) | 11 (100%) | 11 (91.7%) | 5 (100%) | 14 (100%) | 14 (100%) | 85 (93.4%) |
| Female | 0 | 2 (5.3%) | 0 | 2 (15.4%) | 3 (21.4%) | 0 | 0 | 1 (8.3%) | 0 | 0 | 0 | 6 (6.6%) |
| Ethnicity |  |  |  |  |  |  |  |  |  |  |  |  |
| Hispanic or Latino | 4 (30.8%) | 9 (23.7%) | 2 (50.0%) | 3 (23.1%) | 0 | 2 (50.0%) | 5 (45.5%) | 4 (33.3%) | 0 | 1 (7.1%) | 1 (7.1%) | 18 (19.8%) |
| Not Hispanic or Latino | 9 (69.2%) | 29 (76.3%) | 2 (50.0%) | 10 (76.9%) | 14 (100%) | 2 (50.0%) | 6 (54.5%) | 8 (66.7%) | 5 (100%) | 13 (92.9%) | 13 (92.9%) | 73 (80.2%) |
| Race |  |  |  |  |  |  |  |  |  |  |  |  |
| American Indian or Alaska Native | 0 | 0 | 0 | 0 | 0 | 0 | 0 | 0 | 0 | 0 | 0 | 0 |
| Asian | 0 | 1 (2.6%) | 0 | 1 (7.7%) | 0 | 0 | 0 | 0 | 0 | 0 | 0 | 1 (1.1%) |
| Black or African American | 1 (7.7%) | 1 (2.6%) | 0 | 1 (7.7%) | 0 | 1 (25.0%) | 0 | 0 | 0 | 0 | 1 (7.1%) | 3 (3.3%) |
| Native Hawaiian or Other Pacific Islander | 0 | 0 | 0 | 0 | 0 | 0 | 0 | 0 | 0 | 0 | 0 | 0 |
| White | 11 (84.6%) | 35 (92.1%) | 3 (75.0%) | 11 (84.6%) | 14 (100%) | 3 (75.0%) | 10 (90.9%) | 12 (100%) | 5 (100%) | 14 (100%) | 13 (92.9%) | 85 (93.4%) |
| Other | 1 (7.7%) | 1 (2.6%) | 1 (25.0%) | 0 | 0 | 0 | 1 (9.1%) | 0 | 0 | 0 | 0 | 2 (2.2%) |
| Not Reported | 0 | 0 | 0 | 0 | 0 | 0 | 0 | 0 | 0 | 0 | 0 | 0 |

Abbreviations: Q1 = 25^th^ percentile; Q3 = 75^th^ percentile, SD = standard deviation, Max = maximum, Min = minimum

**Supplemental Table 2: Total Opioid Consumption Through 72 Hours in Morphine Equivalent Dose, All Opioids, Full Analysis Set**

|  |  |  | **Cohort 1** | | | **Cohort 2** | | | **Cohort 3** | | |
| --- | --- | --- | --- | --- | --- | --- | --- | --- | --- | --- | --- |
| **Time Period** | **Pooled Placebo (N=13)** | **Pooled Naropin (N=40)** | **Placebo (N=4)** | **Naropin (ropivacaine HC1) (N=14)** | **10 mL of 2% CPL-01 (N=14)** | **Placebo (N=4)** | **Naropin (ropivacaine HC1) (N=12)** | **20 mL of 2% CPL-01 (N=12)** | **Placebo (N=5)** | **Naropin (ropivacaine HC1) (N=14)** | **30 mL of 2% CPL-01 (N=14)** |
| **0 - 24 Hours** |  |  |  |  |  |  |  |  |  |  |  |
| Mean (SD) | 9.19 (9.284) | 7.58 (12.403) | 9.00 (6.868) | 8.14 (16.239) | 2.64 (6.122) | 15.25 (13.301) | 7.04 (9.794) | 3.50 (5.556) | 4.50 (5.123) | 7.46 (10.771) | 2.86 (4.258) |
| Median (Q1, Q3) | 10.00 (0.00, 13.00) | 0.00 (0.00, 12.75) | 10.25 (3.75, 14.25) | 0.00 (0.00, 9.00) | 0.00 (0.00, 0.00) | 15.00 (5.00, 25.50) | 0.00 (0.00, 17.50) | 0.00 (0.00, 7.25) | 2.50 (0.00, 10.00) | 1.25 (0.00, 17.50) | 0.00 (0.00, 5.00) |
| Min, Max | 0.0, 31.0 | 0.0, 60.0 | 0.0, 15.5 | 0.0, 60.0 | 0.0, 20.0 | 0.0, 31.0 | 0.0, 24.5 | 0.0, 15.0 | 0.0, 10.0 | 0.0, 31.0 | 0.0, 10.0 |
| **0 - 72 Hours** |  |  |  |  |  |  |  |  |  |  |  |
| Mean (SD) | 14.58 (17.037) | 15.53 (28.815) | 14.00 (10.352) | 17.39 (38.158) | 2.64 (6.122) | 25.25 (26.352) | 11.75 (17.644) | 4.75 (8.343) | 6.50 (8.588) | 16.89 (27.433) | 7.93 (13.019) |
| Median (Q1, Q3) | 12.50 (0.00, 20.50) | 0.00 (0.00, 23.75) | 16.50 (6.25, 21.75) | 0.00 (0.00, 19.00) | 0.00 (0.00, 0.00) | 20.00 (6.25, 44.25) | 1.25 (0.00, 23.75) | 0.00 (0.00, 7.25) | 2.50 (0.00, 10.00) | 1.25 (0.00, 36.00) | 0.00 (0.00, 15.00) |
| Min, Max | 0.0, 61.0 | 0.0, 143.0 | 0.0, 23.0 | 0.0, 143.0 | 0.0, 20.0 | 0.0, 61.0 | 0.0, 53.5 | 0.0, 25.0 | 0.0, 20.0 | 0.0, 83.0 | 0.0, 42.5 |
| **24 - 72 Hours** |  |  |  |  |  |  |  |  |  |  |  |
| Mean (SD) | 5.38 (8.282) | 7.95 (17.453) | 5.00 (3.536) | 9.25 (22.759) | 0.00 (0.000) | 10.00 (13.693) | 4.71 (9.139) | 1.25 (3.108) | 2.00 (4.472) | 9.43 (17.644) | 5.07 (9.621) |
| Median (Q1, Q3) | 2.50 (0.00, 7.50) | 0.00 (0.00, 5.75) | 6.25 (2.50, 7.50) | 0.00 (0.00, 6.50) | 0.00 (0.00, 0.00) | 5.00 (1.25, 18.75) | 0.00 (0.00, 3.75) | 0.00 (0.00, 0.00) | 0.00 (0.00, 0.00) | 0.00 (0.00, 12.50) | 0.00 (0.00, 10.00) |
| Min, Max | 0.0, 30.0 | 0.0, 83.0 | 0.0, 7.5 | 0.0, 83.0 | 0.0, 0.0 | 0.0, 30.0 | 0.0, 29.0 | 0.0, 10.0 | 0.0, 10.0 | 0.0, 52.0 | 0.0, 32.5 |

Abbreviations: Q1 = 25^th^ percentile; Q3 = 75^th^ percentile; Max = maximum; Min = minimum; SD = standard deviation

**Supplemental Table 3: Overview of Treatment Emergent Adverse Events, Safety Analysis Set**

|  | | | **Cohort 1** | | | **Cohort 2** | | | **Cohort 3** | | |
| --- | --- | --- | --- | --- | --- | --- | --- | --- | --- | --- | --- |
|  | **Pooled Placebo (N=13)** | **Pooled Naropin (N=40)** | **Placebo (N=4)** | **Naropin (ropivacaine HC1) (N=14)** | **10 mL of 2% CPL-01 (N=14)** | **Placebo (N=4)** | **Naropin (ropivacaine HC1) (N=12)** | **20 mL of 2% CPL-01 (N=12)** | **Placebo (N=5)** | **Naropin (ropivacaine HC1) (N=14)** | **30 mL of 2% CPL-01 (N=14)** |
| Total Number of TEAEs | 26 | 65 | 10 | 25 | 18 | 7 | 20 | 35 | 9 | 20 | 27 |
| Number (%) of Subjects Reporting at Least One: |  |  |  |  |  |  |  |  |  |  |  |
| TEAE | 10 (76.9%) | 29 (72.5%) | 4 (100%) | 8 (57.1%) | 9 (64.3%) | 3 (75.0%) | 10 (83.3%) | 10 (83.3%) | 3 (60.0%) | 11 (78.6%) | 10 (71.4%) |
| TEAE by Severity^[1]^ |  |  |  |  |  |  |  |  |  |  |  |
| Mild | 8 (61.5%) | 18 (45.0%) | 4 (100%) | 5 (35.7%) | 6 (42.9%) | 2 (50.0%) | 5 (41.7%) | 7 (58.3%) | 2 (40.0%) | 8 (57.1%) | 4 (28.6%) |
| Moderate | 2 (15.4%) | 11 (27.5%) | 0 | 3 (21.4%) | 3 (21.4%) | 1 (25.0%) | 5 (41.7%) | 2 (16.7%) | 1 (20.0%) | 3 (21.4%) | 5 (35.7%) |
| Severe | 0 | 0 | 0 | 0 | 0 | 0 | 0 | 1 (8.3%) | 0 | 0 | 1 (7.1%) |
| TEAE by Relationship^[2]^ |  |  |  |  |  |  |  |  |  |  |  |
| Suspected | 0 | 6 (15.0%) | 0 | 4 (28.6%) | 0 | 0 | 1 (8.3%) | 0 | 0 | 1 (7.1%) | 0 |
| Not Suspected | 10 (76.9%) | 23 (57.5%) | 4 (100%) | 4 (28.6%) | 9 (64.3%) | 3 (75.0%) | 9 (75.0%) | 10 (83.3%) | 3 (60.0%) | 10 (71.4%) | 10 (71.4%) |
| Treatment-Emergent Opioid-Related AE^[3]^ | 8 (61.5%) | 17 (42.5%) | 3 (75.0%) | 4 (28.6%) | 6 (42.9%) | 2 (50.0%) | 5 (41.7%) | 9 (75.0%) | 3 (60.0%) | 8 (57.1%) | 7 (50.0%) |
| Treatment-Emergent Potential  LAST-related AESI^[4]^ | 0 | 10 (25.0%) | 0 | 4 (28.6%) | 0 | 0 | 4 (33.3%) | 1 (8.3%) | 0 | 2 (14.3%) | 4 (28.6%) |
| TEAE Leading to Study Discontinuation | 0 | 1 (2.5%) | 0 | 1 (7.1%) | 0 | 0 | 0 | 0 | 0 | 0 | 0 |
| TEAE Causing Death | 0 | 0 | 0 | 0 | 0 | 0 | 0 | 0 | 0 | 0 | 0 |
| Total Number of TESAEs | 0 | 0 | 0 | 0 | 0 | 0 | 0 | 2 | 0 | 0 | 0 |
| Number (%) of Subjects Reporting at Least One: |  |  |  |  |  |  |  |  |  |  |  |
| TESAE | 0 | 0 | 0 | 0 | 0 | 0 | 0 | 1 (8.3%) | 0 | 0 | 0 |
| TESAE by Severity^[1]^ |  |  |  |  |  |  |  |  |  |  |  |
| Mild | 0 | 0 | 0 | 0 | 0 | 0 | 0 | 0 | 0 | 0 | 0 |
| Moderate | 0 | 0 | 0 | 0 | 0 | 0 | 0 | 0 | 0 | 0 | 0 |
| Severe | 0 | 0 | 0 | 0 | 0 | 0 | 0 | 1 (8.3%) | 0 | 0 | 0 |
| TESAE by Relationship^[2]^ |  |  |  |  |  |  |  |  |  |  |  |
| Suspected | 0 | 0 | 0 | 0 | 0 | 0 | 0 | 0 | 0 | 0 | 0 |
| Not Suspected | 0 | 0 | 0 | 0 | 0 | 0 | 0 | 1 (8.3%) | 0 | 0 | 0 |

Abbreviations: AE = adverse event; AESI = adverse event of special interest; LAST = Local Anesthetic Systemic Toxicity; MedDRA = Medical Dictionary for Regulatory Activities; TEAE = treatment-emergent adverse event; TESAE = treatment-emergent serious adverse event

[1] Subjects reporting more than one AE were counted only once using the highest severity.

[2] Subjects reporting more than one AE were counted only once using the closest relationship to study drug. Not related events included those reported as “Not Suspected” causality to study drug; related events included those reported as “Suspected” causality to study drug.

[3] Opioid-related AEs included AEs with the following MedDRA preferred terms: nausea, vomiting, constipation, pruritus, somnolence, urinary retention, and respiratory depression.

^[4]^ Potential LAST-related AESIs included AESIs with the following MedDRA preferred terms, which were based on the categories assessed in the LAST symptom assessment: paraesthesia oral, dysgeusia, tinnitus, vision blurred, visual impairment, tremor, muscle twitching, dizziness, seizure, bradycardia, arrhythmia, hypotension, cardiac arrest, and respiratory arrest.
